# Prospective cohort data quality assurance and quality control strategy and method: Korea HIV/AIDS Cohort Study

**DOI:** 10.4178/epih.e2020063

**Published:** 2020-09-04

**Authors:** Soo Min Kim, Yunsu Choi, Bo Youl Choi, Minjeong Kim, Sang Il Kim, Jun Young Choi, Shin-Woo Kim, Joon Young Song, Youn Jeong Kim, Mee-Kyung Kee, Myeongsu Yoo, Jeong Gyu Lee, Bo Young Park

**Affiliations:** 1 Department of Applied statistics, Yonsei University College of Commerce and Economics, Seoul, Korea; 2 Department of Statistics and Data Science, Yonsei University College of Commerce and Economics, Seoul, Korea; 3 Institute for Health and Society, Hanyang University, Seoul, Korea; 4 Department of Preventive Medicine, Hanyang University College of Medicine, Seoul, Korea; 5 Division of Infectious Disease, Department of Internal Medicine, Seoul St. Mary’s Hospital, College of Medicine, The Catholic University of Korea, Seoul, Korea; 6 Department of Internal Medicine and AIDS Research Institute, Yonsei University College of Medicine, Seoul, Korea; 7 Department of Internal Medicine, Kyungpook National University School of Medicine, Daegu, Korea; 8 Division of Infectious Diseases, Department of Internal Medicine, Korea University College of Medicine, Seoul, Korea; 9 Division of Infectious Disease, Department of Internal Medicine, Incheon St. Mary’s Hospital, College of Medicine, The Catholic University of Korea, Incheon, Korea; 10 Division of Viral Disease Research Center for Infectious Disease Research, Korea National Institute of Health, Cheongju, Korea

**Keywords:** HIV/AIDS, Quality control, Cohort studies, Data adjustment, Data quality, Data accuracy

## Abstract

**OBJECTIVES:**

The aim of effective data quality control and management is to minimize the impact of errors on study results by identifying and correcting them. This study presents the results of a data quality control system for the Korea HIV/AIDS Cohort Study that took into account the characteristics of the data.

**METHODS:**

The HIV/AIDS Cohort Study in Korea conducts repeated measurements every 6 months using an electronic survey administered to voluntarily consenting participants and collects data from 21 hospitals. In total, 5,795 sets of data from 1,442 participants were collected from the first investigation in 2006 to 2016. The data refining results of 2015 and 2019 were converted into the data refining rate and compared.

**RESULTS:**

The quality control system involved 3 steps at different points in the process, and each step contributed to data quality management and results. By improving data quality control in the pre-phase and the data collection phase, the estimated error value in 2019 was 1,803, reflecting a 53.9% reduction from 2015. Due to improvements in the stage after data collection, the data refining rate was 92.7% in 2019, a 24.21%p increase from 2015.

**CONCLUSIONS:**

Despite this quality management strategy, errors may still exist at each stage. Logically possible errors for the post-review refining of downloaded data should be actively identified with appropriate consideration of the purpose and epidemiological characteristics of the study data. To improve data quality and reliability, data management strategies should be systematically implemented.

## INTRODUCTION

Longitudinal data are obtained from repeated investigation of specific factors through a long-term follow-up of the same subject. Cohort data, a type of longitudinal data, are an epidemiological study design that compare the incidence or mortality in two groups through long-term follow-ups of a population exposed to a specific risk factor and another that was not exposed which have the advantage that yields a clear temporal precedence relationship between cause and effect.

Bias in cohort studies includes a volunteer bias caused by differences in characteristics between those who voluntarily agreed to participate in the study and those who did not, a follow-up loss bias resulted by death or dropout during the study, an ascertainment bias led by different investigation process of disease information between the exposed and non-exposed group, Hawthorne effect caused by changes in the subject’s behavior by repeated measurement of risk factors, and a time bias affected by changes in diagnostic criteria or subject’s personal factors depending on the follow-up period. Other biases that are also common in most research designs include a non-response bias where there is non-respondents in survey questions, an interviewer bias caused by information bias in the process of investigating with the interviewer, and a measurement bias. In addition, errors may occur unexpectedly and accidentally in the process of conducting research [1]. Thus, it is necessary to perform appropriate quality management in the entire process from pre-phase of the data collection to the time when data are collected.

Quality control is of utmost importance in conducting any study, and the integrity of the study results is determined by the quality of the collected data. If proper quality control is not performed to utilize cohort data, power (1-β) may decrease while type 1 error (α) may increase [2]. Main factors affecting the quality of research data include completeness, accuracy, and timeliness [3], and accurate input of data, monitoring of input data, and data cleaning are required construction for these factors [2,4-7].

According to domestically reported data, the number of surviving infected people excluding those who died by 2018 was 12,991 (25.3/10,000), and the incidence of acquired immune deficiency syndrome (AIDS) per 100,000 population by 2017 was 0.3, which was lower than the Organization for Economic Cooperation and Development average [8,9]. The Korean HIV/AIDS cohort study was established to understand the natural course and epidemiological and clinical characteristics of human immunodeficiency virus (HIV)-infected individuals and patients in Korea from initial diagnosis to AIDS onset and death. In this study, only participants with confirmed HIV positive by the Western blot test and voluntarily consented participated, whom are Korean and over the age of 18 [10]. A total of 16,830 case report forms (CRF) of 1,539 people were collected until October 2019, and the general characteristics, socioeconomic factors, comorbidities (AIDS-related, non AIDS-related), history of antiretroviral therapy (ART) and reasons for termination were measured repeatedly every 6 months [10,11]. These collected data are used by researchers, participating in the Korea HIV/AIDS Cohort Study to conduct intermediary studies related to HIV/AIDS. The purpose of the study is to establish the basis for treatment and prevention methods that are helpful to patients in the clinical field, and research results with poor quality management are difficult to apply to the real world.

In order to test hypothesis of HIV studies and derive high quality research results, proper quality management is required. In this study, based on the existing data quality management strategy, customized quality management methods that consider the characteristics of domestic HIV/AIDS cohort studies was applied to data from the past (the 10th study in 2015) and recent (the 14th study in 2019). The rate of data cleaning was compared to objectively assess the effects of data quality management.

## MATERIALS AND METHODS

### Epidemiology and data center

The HIV/AIDS Cohort Study in Korea has an epidemiological data center that collects data and manages the quality of data from the department of infectious diseases in 21 hospitals nationwide. In the epidemiological data center, there are an epidemiological team in charge of investigation-related consent, a quality management team in charge of data cleaning, and a statistical analysis team that supports research analysis [10].

### Data resource

The Korea HIV/AIDS Cohort Study uses an electronic survey for subjects who have voluntarily consented to repeat measurements every 6 months and collects data in real time from the 21 hospital. The first survey was started in 2006, and by 2016, 5,795 surveys from 1,442 subjects were collected (Supplementary Material 1).

### Data quality assurance and quality control protocol

**Figure 1.**
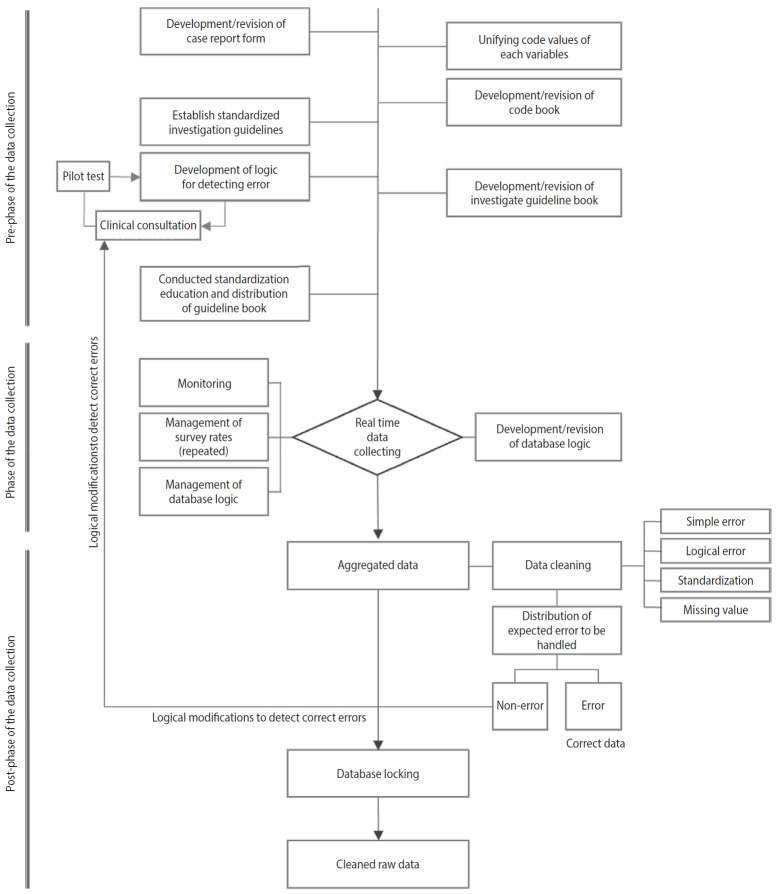
The process of data quality control.

The entire process of the Korea HIV/AIDS cohort research data quality management strategy program is largely divided into prephase of data collection, phase of data collection, and post-phase of data collection. Specific strategies, types, management cycle, methods, and possible errors when unexecuted of each phase were presented. In the pre-phase of data collection, Supplementation and revision of CRF, unifying code values, development of logic for detecting errors, education and development of standardized investigation guidelines were performed. In the phase of the data collection, possible errors at the time of data input were minimized through real-time monitoring and management of repeated CRF rate and database (DB) query language. In the post-phase of data collection, data cleaning was performed using the developed logic for detecting errors. The accuracy of the data was improved through standardization of narrative items and resurvey, which minimize missing values. In all phases, data review included confirmation by infectious disease specialists. In the cleaning process to derive the expected errors to be handled in the data, two or more epidemiological statistic researchers conducted a duplicate review. Furthermore, during the review process of checking if the data have been correctly entered into the DB, the epidemiological data center and DB manager of Korea Centers for Disease Control and Prevention (KCDC) conducted a duplicate review (Figure 1 and Table 1).

If there are many missing values for important questions during the analysis of studies that used the data, those values are replaced with other values such as using the formula, internal or external data to increase the completeness of the data. If there are still missing values even after quality control in the pre-phase and phase of data collection, the values are estimated if possible, and the steps for estimation are as follows:

#### Replacement using a formula

If the date variables were partially missing, the survey date was used to replace the following equation:

$$\mathrm{New}\;\mathrm{HIV}\;\mathrm{diagnosis}\;\mathrm{month}\;(d)\;=\;\mathrm{survey}\;\;\mathrm{month}\;(d)\;-\;\frac{\left(\mathrm{survey}\;\mathrm{month}\;(d)-1\right)}{2}$$

#### Replacement using internal data

If there are no data at a specific time in the existing data, these are replaced with the data at the nearest time within 90 days before or after the specific time. If the results of the immunoassay at the time of diagnosis or at the starting date of initial ART are missing, the tests results at the time of diagnosis or at the closest time before and after the starting date of initial ART are used to replace the data. Replacement rate increased as the permitted period before and after the diagnosis was increased. However, considering the 6 months period of repeated investigation, it was defined as 3 months.

Blood test date - HIV diagnosis date (initial ART date) ≤ 3 mo

#### Replacement using external data

If the values for date of HIV positive diagnosis, date of death, and path of infection (transfusion, blood products) were missing, the data contained in the HIV/AIDS epidemiology survey by KCDC were used as replacement.

### Results of real data application for data quality management strategy

Among the data quality management strategies developed as described above, the quality management method at the post-phase of data collection was applied to data from the 10th and 14th of the HIV/AIDS cohort data, and the rate of data cleaning was compared. Three factors are required to calculate the rate of data cleaning. First, expected errors to be handled, which are estimated to be errors by the developed logic, are required. In addition, corrected errors, which are refined after asking each hospitals to check the expected errors to be handled and non-errors, are values initially classified as errors by logic, but are found as non-error values after checking the medical record. These three factors are used to calculate the rate of date cleaning, which is defined as the percentage of corrected error values and non-error values (sum of corrected errors and non-errors, numerator) compared to the number of the expected errors to be handled requested for confirmation to the hospitals (denominator). Thus, the rate of data cleaning indicates the rate at which the estimated errors that expected by logic is corrected, and higher rate of data cleaning (closer to 100) indicates that more errors could be verified.

$$\mathrm{Rate}\;\mathrm{of}\;\mathrm{data}\;\mathrm{cleaning}\;(\%)\;=\;\frac{\mathrm{corrected}\;\mathrm{errors}\;+\;\mathrm{non}-\mathrm{errors}}{\mathrm{expected}\;\mathrm{errors}\;\mathrm{to}\;\mathrm{be}\;\mathrm{handled}}\;\times \;100$$

### Ethics statement

The purpose of this study is the same as the Korea HIV/AIDS Cohort Study, and it is exempt from research ethics review because no additional data or invasive sample collection has been made to the subject for the study.

## RESULTS

After refining approximately 29 million data consisting of 5,795 CRFs and 5,027 variables through the data quality management strategy, the expected errors in the 14th study was 1,803, which was reduced by 53.9% compared to the expected errors of the 10th study. This result was obtained by systematically performing unifying code values, education of standardized investigation guidelines, and revision and supplementation of research documents at the pre-phase of data collection and DB monitoring and DB logic development at the phase of data collection. Systemic data quality management strategies were not established prior to the derivation of the expected errors for the 10th study. Thus, constructed quality management procedures were not performed, and only education of standardized investigation guidelines and DB monitoring by new clinical research coordinator (CRC) were done temporarily. However, as systemic procedures were established, it was possible to drastically reduce the sporadic expected errors by monitoring all collected CRF in real time and dividing the periodic education of standardized investigation guidelines into regular, occasional and supplementary education.

Despite the systemic quality management strategy up to the point of data collection, missing values were found in the postphase of data collection due to the difficulty of checking the medical treatment and prescription records at other hospitals or the subject’s denial to respond. Missing values in repeatedly measured data can affect the research results. Thus, a method to compensate the missing values was required. Previous studies have used the statistical method of imputation. However, this method is simply based on mathematical and statistical theories and is limited in that the clinical characteristics are not sufficiently considered. In this study, this was compensated by replacing the missing values of CD4 or HIV RNA at the time of diagnosis or at the starting date of initial ART with test results within 1 month or 1 year before or after diagnosis. As a result, CD4 and HIV RNA result values increased from 38.8% and 35.1%, respectively, with test results within 1 month, whereas increased to 73.7% and 71.0% with test results expanded to 1 year. When the immunological test results were defined based on the starting date of initial ART, the results were the same (Table 2). In the case of HIV diagnosis date, 11.9% were missing, of which 11.5% could be replaced by using the HIV/AIDS epidemiology survey by KCDC, and the date of diagnosis of the remaining 0.4% could not be confirmed in both DBs. As such, if data at a specific time point are missing, internal or external data can be used to replace the missing values, which increase the completeness of the data for research purposes.

Furthermore, despite the implementation of the advanced systemic quality management strategy, errors remain when the data are downloaded. This is derived as expected errors that require handling the use of logic for data cleaning in the post-phase of data collection. In the 10th study, data collected between December 2006 and December 2015 were cleaned to derive 3,914 estimated errors, of which 2,051 errors excluding 629 actual values were corrected. The data were cleaned with a 68.5% rate of data cleaning. In the 14th study, data collected from December 2006 to July 2019 were cleaned to derive 1,803 estimated errors, of which 1,274 errors excluding 397 non-errors were corrected. The data were cleaned with a 92.7% rate of data cleaning.

Based on the rate of data cleaning, the quality of data in the 14th study increased by approximately 24.2% compared to the 10th study (Table 3). The Korea HIV/AIDS Cohort Study cleans the data collected during the same period twice through the processes. Once the rate of date cleaning exceeds 90%, the quality of data is thought to be distributable, and the DB of the data refined during the period is locked, so that the data cannot be arbitrarily modified. The locked data can only be modified by the person in charge of data management, and every revision history is recorded to maintain data quality. The cohort operating committee decides if the cleaned data are eligible for distribution. If the data is fit for distribution, internal and external researchers are provided with the data in accordance with the “Regulations on the Procedures for Disclosure of Raw Data from KCDC.” Internal researchers of the cohort submit research proposals to the integrated disease and health system (http://is.cdc.go.kr). Once evaluation and approval from the cohort operating committee are received, the data are distributed in accordance with the regulations. In the cases of external researchers, once the research plan is submitted to the integrated disease and health system, a research plan evaluation committee is held. The data can only be used after the final approval of the head of the Viral Disease Research Division at the Centers for Disease Control and Prevention, based on the evaluation results. After adjusting the usage schedule, the data can be analyzed in the data analysis room for academic research of the Virus Disease Research Division of Centers for Disease Control and Prevention. The analyzed results may be taken out of the room after review and approval. The results of data use must comply with the regulations for research outcomes of KCDC. More information is provided by KCDC integrated disease and health system.

## DISCUSSION

In order to utilize data in research and improve the quality of life of HIV/AIDS infected individuals in Korea, effort of all participants in data collection and cleaning are required. It is important to use the data to proceed with research; however, accurate data must be preceded for accurate analysis. In the Korea HIV/AIDS Cohort Study, the first cohort-customized data quality management strategy that reflects the characteristics of the cohort subjects and data were established, and a method to increase the completeness of the cohort data was suggested. Previous studies showed that most cohort data quality management strategies were implemented at the phase of data collection. However, in this study, the errors between various variables and sequence that were difficult to identify in previous phases were intensively derived by finding and correcting logical errors. These logical errors, which consider clinical and epidemiological characteristics of the disease, were derived in not only the phase of data collection, but also the post-phase of data collection as well. Moreover, a logical error that combines the clinicepidemiological evidence and statistical method of HIV/AIDS was applied to the cohort data. Thus, it is possible to extract and refine, in advance, the error values that are not derived as a simple error on computational basis and that contract the clinic-epidemiological hypothesis. In fact, an error may occur when defining a subject by using multiple items in combination, depending on the research topic or when creating a research variable based on the operational definition of the researcher using previously collected data. Even though the researcher may spot these errors during the process of research, the data cannot be directly verified, and it limits the use of that research. Moreover, if the research is continued without identifying these errors, bias may be introduced in the interpretation of the results. Therefore, logical errors need to be developed in consideration of the purpose of collecting research data, clinic-epidemiological characteristics of the disease, and characteristics of the subject.

The quality management strategy in this study was composed of systematic steps, and each step contributed to data quality management in a complex manner. Therefore, it is important that each step is not omitted and is executed properly. For example, if the pre-phase of data collection, such as revising CFR, training CRC, and development of various guidelines is omitted, it is difficult to apply the changing HIV/AIDS epidemiological characteristics to research. If the format for data collection and use is not standardized, data collection and use are restricted. Furthermore, if the process of phase of data collection is omitted, it is difficult to identify errors in electronic CRF collected in real time. The reliability and validity of the collected data will decrease when problems such as incorrect input and download errors are not resolved due to an error in the electronic CRF. In addition, if data cleaning in post-phase of the data collection is omitted, errors occurring between multiple sequence and questions that were not confirmed in the previous phases cannot be identified.

As shown in this study, the each phase of data quality management strategy reduced the estimated error value by improving the pre-phase and phase of data collection in the 14th study compared to the 10th study and increased the rate of data cleaning through improvement of the post-phase of data collection. Until now, there has been no standardized term to describe results such as rate of data cleaning as a qualitative review method or strategy using epidemiological research data. Studies using clinical data focused only on the flow of data cleaning processes. There were no studies on the detailed steps and results of the cleaning processes. The 68.4% rate of data cleaning indicates that 684 estimated errors have been corrected out of 1,000 errors, while the 316 errors still remain. An increase in the rate of data cleaning shows that the remaining estimated errors have decreased, suggesting that the reliability of the data has improved compared to before.

The quality of data also affects the results of research analysis. It is important to systemically implement data quality management strategies in the future for better quality data and reliable research results. Despite such systematic and complex cleaning processes, there is a limitation to handling all errors and missing values. Due to the characteristics of cohort data, it is necessary to increase the tracking rate to reduce missing values. In addition, errors at the time of entering electronic CRF need to be fundamentally prevented and processing after data collection need to be minimized through several steps. Even if future technology advancements may allow the use of data query language to prevent errors at the time of entering electronic survey, post-review of downloaded data must be performed. In addition to the developed logical errors, potential errors may exist, and cleaned data is not perfect data without any errors. Therefore, steady development and supplementing procedures against logical errors are required.

## Figures and Tables

**Table 1.** Strategy of data management in Korea HIV/AIDS Cohort Study

Step	Strategy	Type	Management period	Method	Possible error when unexecuted
Pre-phase of the data collection	Development and revision of CRF	QA	Annually (if necessary)	During the survey process occasionally collect the opinion of occurrence situation after then development and apply at the next year	Failure to apply changing AIDS dynamics trends and continued collection of data using incorrect surveys limits the research topic
	Unifying code values of each variables	QA	Annually (if necessary)	Code values are defined to match the code values of the developed CRF, DB, and downloaded data	It is difficult to derive simple errors, and there is a limitation in using data
	Establish standardized investigation guidelines	QA	Annually (if necessary)	Discuss with clinical experts about investigation guidelines of each questionnaire	Collected data by multiple researchers in multi-institution may not be combined as the same definition
	Development of logic for detecting errors	QC	Occasionally	Develop logical errors based on clinical and epidemiological facts, including those that differ from the developed guidelines or are out of the code value	There are limitations in the study due to inconsistent relationship errors based on clinical and epidemiological evidence
	Education and distribution of standardized investigation guidelines	QA	Annually	Annually at the start of the study (regular), when new researchers are hired (occasional), and when the survey is changed during the study or the expected error value is high (supplementary) conduct standardization training with distribution of standardized investigation guidelines	Each investigator has a different understanding of the survey guidelines (documents), which can lead to errors
Phase of the data collection	DB monitoring	QA	Occasionally	Review the data collected in the DB in real time and feedback it to the investigator	Since the error occurred at the time of data entry could not be corrected immediately, additional work is required
	Management of repeated survey rates	QC	Quarterly	Confirmed and distributed of the tracking and loss to follow-up rates	There is no management of participants' dropouts
	Management of DB logic	QC	Occasionally	Developing DB logic to prevent possible errors during the DB input confirmed through real-time monitoring	Monitoring and data cleaning are difficulties when the DB cannot block the errors that occur at the time of data entry
Post-phase of the data collection	Raw data cleaning	QC	Annually	Refining within or between the sequence errors including simple errors for all collected data	Various errors may cause errors in the analysis phase or study limitations that subjects are excluded
	Standardization of the descriptive questions	QC	Every 2 yr	Defined as a new variable by standardizing descriptive response values that are difficult to identify and to calculate frequency; Existing variables can be left to compare	The same narrative response value is recognized differently, or the research results are derived differently by the researcher's operational definition
	Re-survey	QC	If necessary	When there is a change in the survey questionnaire or when the error is high, the historical data is re-confirmed through a medical record, a direct review of the participants, or a doctor's review	Incorrect data may affect research results
	Substitution using the external data	QC	Annually	The Centers for Disease Control and Prevention used HIV epidemiological reporting data to replace missing values in some questions	There is a limitation in the use of the research due to missing items in the main questions.
	Revision of the logic for detecting errors	QC	Annually	Revision of the logical error for data cleaning when the expected error value decreases by applying DB logic	Detected inaccurate expected errors due to incorrect logic
	Cleaned raw data usage guidelines	QA	Annually	Develop guidelines for refined data usage method, including CRFs and defined code values	It is difficult to know the data accurately, which affects the results
	Development of the DMP	QA	Annually	Develop and revision of the DMP based on data quality control strategy and progress.	Manage data quality systematically without missing procedures.
	Annual statistical report	-	Annually	Descriptive statistics are calculated annually using refined data	Difficult to identify characteristics of study participants
	Evaluation of the research feasibility and statistical support (internal researchers only)	-	Occasionally	The feasibility of the study was reviewed by reviewing the number of subjects and event cases according to the research topic; Consultation and support are provided for the design and analysis of epidemiological and statistical analysis specialists	Prevent delays in deriving research outcomes as data limitations.

QA, quality assurance; QC, quality control; CRF, case report form; DMP, data management plan; AIDS, acquired immune deficiency syndrome; DB, database; HIV, human immunodeficiency virus.

**Table 2.** Response rate of CD4/viral load by imputation methods

Response rate (%)	Within from diagnosis date		Within from initial ART	
	CD4	HIV RNA	CD4	HIV RNA
30 d	38.8	35.1	64.5	60.1
90 d	59.2	55.7	74.4	70.5
180 d	66.6	64.6	75.7	72.3
270 d	70.9	68.7	76.0	72.6
1 yr	73.6	71.0	76.1	72.9

ART, antiretroviral therapy; HIV, human immunodeficiency virus.

**Table 3.** Improvement of the rate of data cleaning within 5 years

Variables	10th HIV/AIDS Cohort Study	14th HIV/AIDS Cohort Study
Data collection duration (visit)	Dec 2006-Dec 2014	Dec 2006-Jul 2019
Data cleaning duration (mo)	May 2015-Nov 2015	Jul 2019-Nov 2019
Organization	16 hospitals	15 hospitals
Expected errors to be handled^1^	3,914	1,803
Corrected errors^2^	2,051	1,274
Non-errors^3^	629	397
Unconfirmed^4^	19	132
Rate of data cleaning (%)^5^	68.5	92.7

1 Derived value through the data cleaning procedure using the logic.

2 The value identified as an error and corrected.

3 The value was derived as an error, however actual observed value.

4 The error value unverified yet.

5 $\frac{\left(Corrected\;errors\;+\;Non-errors\right)}{Expected\;errors\;to\;be\;handled}\;\times \;100$

## SUPPLEMENTARY MATERIALS

Supplementary Material 1.

Korean version is available at http://www.e-epih.org/.
