# Supplementary material for: Prospective cohort data quality assurance and quality control strategy and method: Korea HIV/AIDS Cohort Study
Source: Epidemiol Health. 2020 Sep 4;42:e2020063. doi: 10.4178/epih.e2020063 (PMC7871148; doi:10.4178/epih.e2020063)
Supplement: Supplementary Material 1. [file epih-42-e2020063-suppl1.pdf]

만성감염질환 코호트 데이터 품질관리 표준화 지침

2019. 12.

한양대학교 예방의학교실

국립암센터

연세의료원 내과학교실

질병관리본부 국립보건연구원 바이러스질환연구과

Ver 2.0

목 차

1. 만성감염질환 코호트 연구 ··· 1
- 1) 목적 ··· 1
- 2) 현황 ··· 2

2. 만성감염질환 코호트 연구 데이터 품질관리 용어 ··· 3
- 1) 조사와 관련된 용어 ··· 3
- 2) 품질관리와 관련된 용어 ··· 5

3. 만성감염질환 코호트 연구 데이터 품질관리 전략 ··· 10
- 1) 코호트 데이터 품질관리 전략의 필요성과 목적 ··· 10
- 2) 코호트 데이터 품질관리 전략과 방법 ··· 10
  - (1) 자료 수집 전 단계 품질관리전략 ··· 11
  - (2) 자료 수집 단계 품질관리전략 ··· 13
  - (3) 자료 수집 후 단계 품질관리전략 ··· 14
- 3) 코호트 연구별 데이터 품질관리 전략 수립 시 주의사항 ··· 15

4. 데이터 품질관리 지침서 작성 기준 ··· 16
- 1) 조사서 및 조사 지침서 ··· 16
- 2) 원시자료이용지침서 ··· 17
- 3) 코드북 ··· 17
- 4) 자료 관리 계획서 ··· 18

5. 연구 담당자 ··· 19
- 1) 만성감염질환 코호트 데이터 품질관리 지침 개발 ··· 19

1 만성감염질환 코호트 연구

1) 목적

만성감염질환은 일반적인 감염질환과 달리 감염 이후 만성 질환과 같이 지속적인 관리와 치료가 필요한 질환을 말한다. 대표적으로 HIV/AIDS, 간염, 인유두종 바이러스(HPV), 그리고 결핵 등이 있는데, 만성감염질환의 관리는 국가의 사회·경제적 손실을 최소화하고 감염인과 국민 전체의 건강한 삶을 보장하기 위해 필수적이다.

국가는 만성감염질환의 효과적인 관리 기반을 마련하기 위해 2006년 HIV/AIDS와 HCV 코호트 연구를 시작으로 현재 5개 코호트 연구를 구축하여 반복조사를 시행하고 있다.

2) 현황

2017년까지 전체 5개 코호트에 참여한 대상자는 7,000여명이다. 질환의 특성이 만성감염질환이라는 점에서 공통점을 가지고 있으나, 실제 바이러스의 특징과 이에 따른 결과 및 예후에 큰 차이가 있어 기본적인 조사항목을 제외하고, 질환별 특징과 관련하여 조사되는 문항과 조사 방식에 따른 변수는 서로 다르다.

조사내용	
**대상병원체**	HIV/AIDS[1], HCV[2], HBV[3], HPV[4], TB[5]
**조사항목**	인구학적 조사, 생활습관 조사, 치료력, 임상검사 등의 공통변수(67개)와 각 질환별 특성에 대한 변수
**수집자원**	혈장, PBMC[6]

표 1 국내 만성감염질환 코호트 연구 조사내용

1) 인간면역결핍바이러스/후천성면역결핍증후군(Human Immunodeficiency Virus/Acquired Immune Deficiency Syndrome)
2) C형 간염 바이러스(Hepatitis C Virus)
3) B형 간염 바이러스(Hepatitis B Virus)
4) 사람인유두종바이러스(Human Papilloma Virus)
5) 결핵(Tuberculosis)
6) 말초혈액단핵세포(Peripheral Blood Mononuclear Cell)

Ver 2.0

각 코호트별 연구의 주요 목적과 구축·운영 현황은 아래 표 2와 같다.

구분	HIV/AIDS	HCV	HPV	HBV	TB
**목적**	AIDS 발병과 사망까지 질병 진전에 미치는 다양한 요인 규명	감염위험인자 분석, 예방·역학, 항바이러스치료의 적용률과 치료 효과 및 예후 규명	질병진전에 관여하는 역학, 바이러스학, 유전학, 면역학적 위험인자 규명	간질환, 간암 이행 위험인자 및 치료제 내성 발생원인 규명	항 결핵제 내성이 치료예후에 미치는 영향을 파악하여 효과적인 결핵 검사, 치료 및 관리법 개발
**시작연도**	2006년	2006년	2009년	2015년	2016년
**참여기관**	22개 의료기관 (감염내과)	7개 의료기관 (소화기내과)	6개 의료기관 (산부인과)	5개 의료기관 (소화기내과)	3개 의료기관 (호흡기내과)
**대상**	18세 이상 HIV 감염 내국인	18세 이상 HCV 항체 양성 내국인	20~60세 HPV 감염 내국인 여성	19세 이상 HBV 감염 내국인	18세 이상 결핵 환자
**조사주기**	6개월	6개월	6개월	6개월	2/4/8주, 6개월
**최종질환**	기회감염, 관련/정의질환, 대사합병증, AIDS 발병, 사망	지속감염, 간경화, 간암, 사망	지속감염, CIN Ⅱ,Ⅲ[7]	항바이러스제 치료 효과, 간경화, 간경화 합병증, 간암, 사망	지속감염, 내성현황, 치료예후
**누적 등록자수**	1,524 명	3,231 명	1,694 명	1,125 명	600 명
**조사건수**	15,616 건	24,103 건	6,845 건	4,161 건	3,000 건
**인체유래물**	혈장, PBMC (11,625 건)	혈장, PBMC (3,989 건)	혈장, 혈청, Buffy coat, Cervix cell DNA (2,994 건)	혈장, PBMC (2,183 건)	객담, 균주 (1,452 건)

표 2 국내 5개 만성감염질환 코호트 연구 목적 및 구축·운영 현황(19년 6월 기준)

7) 치료가 필요한 자궁경부이형성증(Moderate/severe dysplasia)

2 만성감염질환 코호트 연구 데이터 품질관리 용어

만성감염질환 코호트 연구는 모두 2개 이상의 기관이 협업하여 조사와 연구를 진행하는 다기관 코호트 연구이다. 다기관 코호트 연구의 특성상 연구 전반에 사용되는 용어에 기관별 차이가 있고, 이는 코호트 연구별 차이로 이어진다. 만성감염질환 코호트 연구에서는 체계적인 데이터 품질관리를 위해 각 연구에서 사용 중인 용어와 정의를 표준화하고, 이를 명시하여 상호간 소통에 오류를 최소화한다.

1) 조사와 관련된 용어

- **연구대상자**

해당 코호트 연구의 등록번호를 부여받아 연구에 참여하는 대상자

코호트	내 용
HIV	Western blot에 의한 HIV 양성 확진된 대한민국 국적의 만 18세 이상 성인 가운데 사업 참여에 대해 자발적으로 동의한 감염인
HCV	HCV 항체 양성 확진 된 대한민국 국적의 만 18세 이상 성인 가운데 사업 참여에 대해 자발적으로 동의한 감염인
HPV	자궁경부세포검사상 비정상 상피세포(ASCUS)이거나 저등급병변(LSIL)로 판독되고, DNA 검사상 HPV 양성인 만 20세 이상 60세 이하의 대한민국 국적의 여성
HBV	Serum HBsAg 양성이 6개월 이상 지속되거나 병력 청취 상 과거력이 확인 된 대상자 가운데 대한민국 국적의 만 19세 이상의 성인 가운데 사업 참여에 동의한 자

- **코호트등록일**

코호트 연구에 등록한 날짜(코호트 사업 참여 동의서 서명일)

- **조사서**

연구대상자를 대상으로 조사 시 사용하는 서식

기반조사서: 연구에 필요한 기본정보와 질병과 관련된 모든 요소를 조사하기 위해 개발된 조사도구

반복조사서: 기반조사 항목 중 시간의 흐름에 따라 변화가 가능하여 반복적으로 조사가 필요한 문항을 위해 개발된 조사도구

종결조사서: 대상자가 더 이상 연구에 참여하지 못하게 된 사유를 조사하고, 경우에 따라 질병 관련 마지막 상태에 대해 조사하기 위해 개발된 조사도구

Ver 2.0

- **기반조사(기반조사서를 이용한 조사)**

코호트 등록 시점 혹은 그 이후 최초의 조사

- **반복조사(반복조사서를 이용한 조사)**

약속된 조사주기에 따라 시행되는 조사

- **종결조사(종결조사서를 이용한 조사)**

사망, 전원, 동의철회, 탈락 등의 사유로 더 이상 연구에 참여하지 않는 상태에서 시행하는 조사로 경우에 따라 사망 시점 마지막 질병 관련 상태에 대해서도 조사

- **조사일**

조사를 시행한 날짜

- **최적 방문조사일**

각 연구에서 정한 반복조사주기에 의해 지난 조사 이후 조사가 권장되는 조사일

- **허용 방문조사 기간**

최적 방문조사일을 기준으로 반복조사를 권장하는 기간

- **탈락**

각 연구에서 정의한 기준에 따라 연속적으로 반복 조사에 실패할 경우 연구에 참여하지 않는 것으로 간주하는 종결 사유의 종류

- **부속 연구(satellite study)**

코호트 연구에 참여하는 대상자 중 특정 조건에 부합(최종질환 발생 등)하고, 조건과 관련한 추가 정보가 필요하여 시행하는 연구

- **부가 연구(additional study)**

연구를 목적으로 현재 코호트에 참여하지 않는 독립적인 집단에 대해 시행하는 연구

2) 품질관리와 관련된 용어

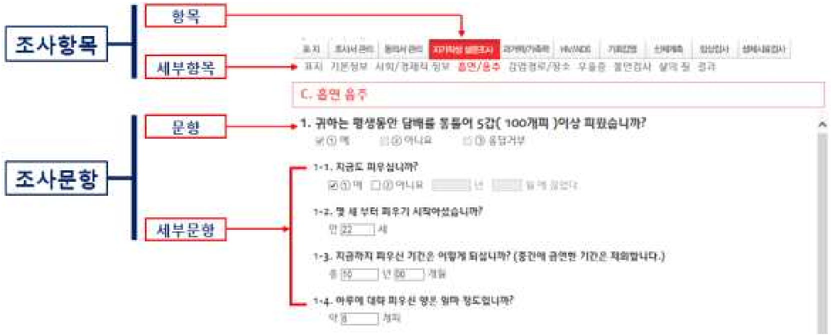

그림 1. 조사항목과 문항의 구분

- **조사항목**

실제 질병과 관련된 요인의 특성을 파악하기 위해 수집하는 설문 범주

- **조사문항**

실제 질병과 관련된 요인의 특성을 파악하기 위해 수집하는 문항(종류)

- **변수**

① 조사변수

조사문항의 특성에 따라 세부 문항과 다운로드 자료 내 문항을 지칭하는 한글변수와 영문변수

② 생성변수

조사된 변수의 응답 값을 활용하여 DB 로직에 의해 자동 계산된 변수

- **원자료**

수집한 자료의 입력오류, 조사 오류 등이 정제되기 이전 단계의 자료

- **원시자료(배포용 자료)**[8]

원자료 정제를 통해 구축된 자료로 분석을 위한 기초 자료

- **조사지침서**

조사 수행 시 표준화된 조사 지침이 명시된 서식

8) http://www.law.go.kr/행정규칙/질병관리본부원시자료공개절차등에관한규정

Ver 2.0

- **코드북**

원자료에 대하여 약속된 코드 값과 변수의 형태, 한글변수/영문변수 명이 명시된 서식

- **원시자료이용지침서**

배포되는 원시자료에 포함된 자료 활용에 사용할 수 있도록 조사서, 변수명, 코드 값을 파악할 수 있는 서식

- **자료 관리 계획서**

자료 질 관리 전반의 수행 계획 및 방법에 대해 명시된 서식

- **추적조사**

코호트 자료 내 수집된 결과와 공공 자료 또는 주요 인물을 통해 직접/전화 조사를 통해 연구 참여 대상자의 최근 상태 및 정보를 추적

- **반복조사율**[9]

반복 조사가 가능한 코호트 등록 대상자[10] 중 최근[11] 반복조사를 시행한 대상자의 비(%)

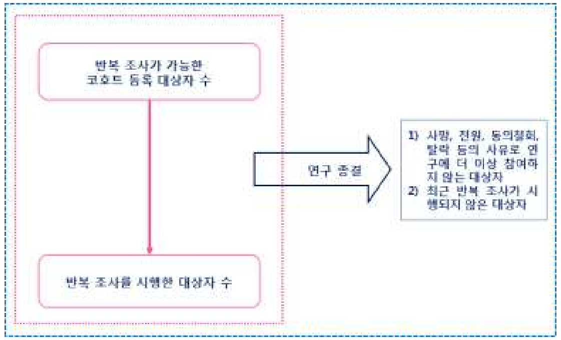

$$\text{반복조사율} = \frac{\text{최근 반복조사를 시행한 대상자 수}}{\text{반복조사가 가능한 대상자 수}} \times 100$$

9) 인구집단의 역학적 특성이 다른 집단에 대한 보건지표를 산출할 때에는 율의 표준화를 통해 집단의 역학적 특성을 보정해주어야만 한다(대한예방의학회. 예방의학과 공중보건학 제 3판. 계축문화사. 2017:108.). 추적관찰을 시행하는 연구에서 초기 연구 집단에 비해 탈락으로 인해 질병 발생 여부를 확인할 수 있는 대상자가 줄어들어 최종 연구 집단에 선택적 선정이 일어나게 되는데 이를 추적관찰 탈락 바이어스(follow-up loss bias)라고 하고(대한예방의학회. 예방의학과 공중보건학 제 3판. 계축문화사. 2017:99.), 환자 코호트의 경우 반복조사가 더 이상 불가능한 대상자로 인해 추적률이 낮아지는 효과를 보정하기 위해 추적이 불가능한 대상자를 제외한 반복조사율을 산출한다.
10) 반복조사가 가능한 코호트 등록 대상자 수=전체 코호트 등록 대상자 수-연구 종결 대상자 수
11) 각 연구에서 정한 객관적 기준(6개월, 12개월 등의 반복조사주기)에 따름

■ **자료 수집 전 단계 품질관리**

표준화된 자료의 수집을 위해 수집 전 수행되어야 할 품질관리 전략

① 조사서 개발/수정/보완

조사에 필요한 정보 수집을 위해 개발된 조사서를 지속적으로 수정/보완하는 단계

② 코드값 일원화 및 코드북 수정/보완

조사서에 근거하여 개발된 Database (DB), 그리고 다운로드 자료 사이에 약속된 코드값은 모두 일치해야하며, 이에 대해 정의된 코드 값은 코드 북에 명시하고 조사서 및 DB 변경시점마다 지속적으로 수정/보완하는 단계

③ 표준화 조사 지침 마련

자료의 신뢰도를 높이기 위해 표준화 조사지침을 규정하고 조사 시 참고할 수 있는 지침서를 개발

④ 논리적 오류(Logic) 개발

예측 가능한 오류를 타당한 표준화 조사 지침과 의학적 근거에 기초하여, 형식상 논리를 통계적으로 추론·도출할 수 있는 문항·회차 간 상호 연관성을 파악하여 오류 예상 값으로 도출 가능한 로직(Logic)을 개발하는 단계

ex. 최초치료시작일은 양성확진일보다 빠를 수 없다.

⑤ 조사원 표준화 교육

마련된 지침에 기반하여 조사 방법 및 자료 수집 시 주의사항, 그리고 DB(e-CRF) 입력 방법과 발생 가능한 오류에 대하여 교육

신규교육: 조사원 변경 시점에 시행하는 표준화 교육

정기교육: 적어도 년 1회 정기적으로 시행하는 표준화 교육(연구시작시점)

보수교육: 예기치 못한 이유로 즉시 교육이 필요한 경우 시행하는 표준화 교육
ex. 조사서 변경, 정제 과정에서 실제 값 보다 오류 예상 값이 더 많이 도출된 경우

⑥ 조사지침서 배포

조사서 사용 방법과 조사 시 주의사항, 조사에 필요한 정보를 포함하고 있으며, 각 연구별로 정해진 주기에 맞춰 조사원에게 배포

■ **자료 수집 단계 품질관리**

다기관 코호트 연구에서 서로 다른 조사원에 의해 수집되면서 발생 가능한 입력 시점 오류를 최소화하기 위해 수행되어야 할 품질관리 전략

① 자료입력관리(모니터링)

실시간으로 수집되는 DB 입력 자료를 주기적으로 환류하여 입력시점 발생 가능한 오류를 검토하여 자료의 질을 관리하는 과정

② 조사율 관리

연구에 참여하는 대상자가 정해진 주기에 맞춰 조사를 시행하고 있는지 확인하고, 산출된 조사율에 대해 각 연구 참여 병원에 공지하여 자료 입력 독려 및 연구 참여자 관리 지표로 사용 가능하도록 함

③ DB 로직 관리

자료입력관리(모니터링)를 통해 확인된 발생 빈도가 높은 입력 시점 오류에 대하여 DB 상 로직을 적용하여 애초에 입력이 불가능하도록 함

■ **자료 수집 후 단계 품질관리**

앞선 두 단계의 정제 과정에서 확인되지 못한 오류를 도출하기 위하여 수집된 자료를 검증하고 연구 성과물 도출을 지원하는 품질관리 전략

① 원자료정제

코호트 연구 시작 이후 직전 년도(~ 12월 31일까지) 수집된 전 자료의 그 상태를 개발된 "논리적 오류(Logic)" 로 오류 예상 값을 도출하고, 의무기록을 참고하여 수정하는 정제 과정으로 이 과정을 통해 정제가 완료된 자료를 '원시자료'라고 정의

■ **자료정제율**

$$\text{자료정제율} = \frac{\text{조사 기관에서 확인 및 수정된 오류 예상 값의 수}}{\text{확인 요청된 오류 예상 값의 수}} \times 100$$

② 서술형문항표준화

조사원 및 연구 참여대상자가 주관에 따라 입력 가능한 기타·서술형문항의 응답 값을 연구에 활용할 수 있도록 표준화하여 새로운 변수로 범주화하는 과정

③ 재조사

오류 가능성이 높아 여러 과정을 통해 참값을 찾는 조사

대상자재조사 연구에 참여하는 대상자에게 직접 설문, 전화 등을 통해 시행하는 재조사

임상재조사 대상자 이외의 의무기록, 주치의 설문 등을 통해 시행하는 재조사

④ 추적조사

코호트 자료의 수집 근거(대상자 대면조사, 의무기록조사) 이외의 관련 국가 자료를 활용하여 통합 자료를 구축하는 정제방법

⑤ 논리적 오류(Logic) 수정/보완

원자료정제 결과에 따라 각 병원에 확인을 마친 오류 예상 값[12] 중 실제 값의 비율이 높은 논리적 오류에 대하여 수정을 진행하며, 오류 값으로 확인된 논리적 오류 중 그 발생 빈도가 높은 건에 대하여 보수 표준화교육을 시행

▪ **오류의 종류**

**단순 오류:**	한 문항 내 코드 값 불일치, 하위문항 누락 등의 오류
**회차 내 오류:**	한 회차 안에서 서로 다른 2개 이상의 문항 사이에 발생할 수 있는 논리적 오류
**회차 간 오류:**	2회 이상의 반복 조사에서 같은 혹은 다른 문항 사이에 복합적으로 발생할 수 있는 논리적 오류
**응답거부/모름/결측:**	- 응답거부(무응답): 조사 대상자가 실제 응답을 원치 않는 경우<br>- 모름: 조사 대상자 혹은 조사자(연구자)가 정확히 모르는 경우<br>- 결측: 응답을 거부한 것인지 모르는 것인지 불확실한 경우 혹은 의무기록 상 정보를 확인할 수 없는 경우

⑥ 원시자료이용지침 마련

정제 전 과정을 통해 구축된 원시자료를 활용하는 데 어려움이 없도록 분석에 용이한 원시자료 이용지침을 마련함

⑦ 자료 관리 계획 개발/수정/보완

년 1회 당해 연도 자료 정제 및 품질관리 결과에 근거하여 차기 연도 자료 관리 계획을 수립하여 지속적인 자료 질 관리를 도모함

⑧ 통계지표산출

원시자료를 활용함에 있어서 자료의 일반적인 특성을 기술통계량으로 제시하여 관련 연구의 근거가 되도록 함

12) 정제 과정을 통해 Logic에 의해 도출된 오류로 의심되는 값으로 이 과정에서 병원 확인 후 실제 값(오류 아님), 오류 값으로 구분된다.

3 만성감염질환 코호트 연구 데이터 품질관리 전략

1) 코호트 데이터 품질관리 전략의 필요성과 목적

(1) 필요성

- 만성감염질환 코호트 연구의 목적은 연구 대상자의 건강증진 및 삶의 질 향상과 더불어 국내 해당 질환 감염인을 위한 효과적인 정책 기반 마련에 있다. 근거 중심의 접근을 위해 다양한 주제에 대한 연구 성과물 도출이 필요하고, 질 높은 연구 성과물 도출을 위해 질 높은 자료가 구축되어야 한다.
- 데이터는 연구자의 편의성과 표준화된 결과 도출을 위해 내려 받는 시점에 상관없이 결과에 차이가 발생하지 않도록 일관성을 유지해야 한다.
- 코호트 연구의 특성상 시간의 흐름에 따른 조사를 위해 시점에 대한 문항이 많다. 시점에 대한 문항에서 오류가 발생하면 코호트의 장점을 활용할 수 없기 때문에, 이를 최소화하기 위하여 문항 간 논리적 상관성을 파악하고 시점뿐만 아니라 의학적 오류에 대한 정제 작업이 필요하다.

(2) 목적

만성감염질환 코호트 연구의 특성을 고려한 데이터 품질관리 전략을 구축하고 이를 기반으로 지속적인 데이터 질 관리를 통해서 질 높은 코호트 데이터를 구축한다.

2) 코호트 데이터 품질관리 전략과 방법

만성감염질환 코호트 연구에서의 데이터 품질관리는 크게 세 단계로 구분하며, 단계별 세부항목은 연구별 특성에 따라 조율이 가능하다. 단계별 세부 품질관리전략은 매 해 연구 종료 시점에 향후 계획을 수립하여 자료 관리 계획서에 기록한다. 체계적이고 지속적인 데이터 품질 관리를 위해서 표준화 지침에 명시된 표준관리기준을 따른다.

(1) 자료 수집 전 단계 품질관리

■ 조사서 개발/수정/보완

관리필요성	해당 질병의 추이를 파악하고, 설문조사 과정에서 발생할 수 있는 문제들을 반영하지 못한 정보의 수집을 막기 위해서는 조사서를 지속적으로 수정 및 보완하여 개발하는 것이 필요하다.
관리 방법	매년 연구 수행 중 조사 시 발생하는 임상적 특성, 상황 등에 대한 의견을 상시 수집하고, 정기 운영위원회와 실무위원회 회의를 통해 개발이 결정된 사항에 대해서는 차기년도 조사서에 적용한다.

■ 코드 값 일원화

관리필요성	개발된 조사서와 입력 시 사용하는 DB 상 전자조사서(e-CRF), 그리고 DB에 수집된 자료를 다운로드 하였을 때 사전에 약속된 코드 값 정의와 동일한 자료가 구축되어야 한다.
관리 방법	조사서가 구축되는 시점부터 모든 자료의 수집과정에서는 약속된 코드 값이 정의될 수 있도록 사전 검토(파일럿 테스트)를 시행하고, 이후 조사서가 수정되는 시점에 지속적으로 일원화 작업을 시행한다.

■ 코드북 개발/수정/보완

관리필요성	조사를 통해 수집된 정보를 객관적인 자료로 구축하기 위해서는 입력된 자료의 저장방식을 사전에 정의해야만 한다.
관리 방법	데이터 정제와 분석 시 데이터 파악이 용이하도록 조사서가 수정될 때마다 수정 및 보완하는 것을 권장한다.

■ 표준화 조사 지침 마련

관리필요성	표준화 된 조사 지침이 없는 경우, 혹은 조사원, 조사 시점에 따라 같은 문항에 대하여 다른 해석으로 조사를 수행할 수 있다. 이는 표준화된 자료 수집을 어렵게 만들기에 사전에 문항별 조사 지침을 마련해야한다.
관리 방법	현장에서 직접 조사를 진행하는 조사원의 의견을 반영하여 각 조사 항목에 대해 임상전문가와 논의 하고 표준화된 조사 지침을 마련하며, 조사서 수정이 있을 때마다 지속적으로 관리한다.

■ 논리적 오류(Logic) 개발

관리필요성	단면 연구 시에는 큰 오류를 확인하기 어려우나, 경시적 자료 분석 시 시간의 선후관계가 맞지 않거나 임상적 오류가 발생할 수 있어 이에 대한 관리가 필요하다.
관리 방법	연구팀은 가능하면 수시로 조사서 문항 별 특성을 고려하여 표준화 조사 지침을 벗어나거나, 지침에는 이상이 없으나 수집된 자료를 분석할 때 오류 예상 값을 도출할 수 있는 방법을 개발한다.

■ 조사원 표준화 교육

관리필요성	조사원들의 연구 목적 및 자료 수집과정에 대한 이해도가 달라 잘못된 데이터가 생산되지 않도록, 표준화 조사 지침과 자료 수집의 전 과정에 대한 교육을 시행하는 것이 필요하다.
관리 방법	신규 연구원 임용시점(신규), 매년 연구 시작시점(정기), 연구 수행 중 조사서 변경 또는 오류 예상 값 도출이 많은 경우(보수) 표준화 교육을 실시한다.

■ 조사 지침서 배포

관리필요성	조사를 수행 할 때 의문이 있는 조사 지침에 대해서는 수시로 확인 가능하도록 하고, 업데이트 된 지침서 내용을 조사 과정에 반영할 수 있도록 조사 지침서를 배포하는 것이 필요하다.
관리 방법	매년 정기 표준화 교육 이후 당해 연도 조사지침서를 책자 또는 전자문서 형태로 배포한다.

(2) 자료 수집 단계 품질관리

■ 자료입력관리(모니터링)

관리필요성	입력 시점에서 발생하는 오류 값을 사후에 처리하게 되면 오류를 수정하는 데 시간차가 발생하거나, 해당 정보의 손실 가능성이 있다.
관리 방법	데이터베이스(DB) 상 입력되는 값을 실시간으로 확인하여 입력 시점에 발생하는 오류 값을 즉시 수정 가능하도록 하며, 해당 내용에 대해서는 관련 연구진들과 전자메일로 공유하도록 한다. 실시간 모니터링이 어려운 경우, 각 연구에서 정한 주기에 따라 연구 참여병원에 직접 방문하여 자료 입력 방법을 점검한다.

■ 조사율 관리

관리필요성	연구에 등록 된 대상자의 탈락을 최소화 하고, 기존 및 신규 대상자를 파악하여 효율적으로 연구 대상자를 관리하기 위해 조사율을 주기적으로 확인하여 관리 할 필요가 있다.
관리 방법	각 연구에서 정한 기준에 따라 정기적으로 조사율을 관리하여 관련 연구진에게 공지하고, 연구 참여대상자 관리 및 조사 자료 입력을 독려한다.

■ DB 로직 관리

관리필요성	자료 입력 시점에서 오류 발생을 줄일 수 있어 자료의 신뢰성을 높일 수 있다. 입력 시점 오류를 줄이면 자료 수집 후 오류를 찾아 수정하는 것보다 빠른 정제작업이 가능하다.
관리 방법	자료 입력 시마다 반복적으로 발생하는 오류의 경우에는 DB 로직을 수정하여 DB상에서 오류 값이 자동으로 수정될 수 있도록 한다.

(3) 자료 수집 후 단계 품질관리

■ 원 자료정제

관리필요성	수집된 자료를 각 회차 별로 나누어 정제하면 문항 또는 회차 간 연관성을 파악하기 어렵기 때문에, 수집된 자료를 시간적 선후관계, 그리고 문항 간 연관성 등을 고려한 정제과정이 필요하다.
관리 방법	매년 수집된 모든 자료에 대해 항목별 단순오류, 회차 내 오류, 회차 간 오류, 결측 값 처리를 모두 확인하도록 한다.

■ 서술형 문항 표준화

관리필요성	대상자의 주관적 응답을 분석 사용할 경우 연구자 정의에 의해 같은 값을 나타내는 응답에 대해서 다르게 해석할 수 있어 이에 대한 표준화 자료를 구축하는 관리 작업이 필요하다.
관리 방법	수집 된 서술형 문항 응답 값이 분석에서 활용 가능 하도록(빈도 산출이 가능하도록) 약어/영문/한글을 고려하여 표준화 하고, 임상 자문위원에 의해 최종 결정된 응답의 경우에는 표준화된 값을 새로운 변수 선택지로 추가할 수 있다.

■ 재조사

관리필요성	문항의 변경, 통계지표가 실제와 다르게 도출되는 경우 특정 문항에 대하여 과거 자료를 모두 확인하는 작업을 시행한다.
관리 방법	운영위원회의 결정에 따라 필요시에는 재조사를 시행한다. 재조사 방법에는 의무기록 확인, 대상자 직접 재조사, 주치의 재조사 등을 통해 과거 자료를 재확인하고, 수정사항은 모두 기록으로 남긴다.

■ 추적조사

관리필요성	더 이상 연구에 참여하지 않아 이후 질병의 발생 혹은 사망을 알 수 없을 때, 국가등록·신고 자료를 이용하여 종결 대상자의 마지막 상태에 대한 신뢰도를 높인다.
관리 방법	사망 이외의 사유로 연구에 참여하지 않는 감염인의 국가신고자료를 활용하여 정확한 자료를 조사하기 힘든 문항에 대하여 재확인을 시행한다. 이미 입력된 자료이더라도 신고 자료와 불일치하는 자료에 대해서 병원 재확인 후 자료를 대치한다.

■ 원시자료이용지침 마련

관리필요성	수정 및 보완된 로직 정보를 반영하여 정제된 자료를 분석할 수 있도록 원시 자료 이용지침을 마련하는 것이 필요하다.
관리 방법	데이터 코딩값에 대한 설명과 함께 연구 진행 과정에서 변경된 조사 문항들에 대해 연구자가 이해 할 수 있도록 조사 문항의 흐름을 확인 할 수 있도록 하는 것이 좋고, 원시자료 변경 시점 마다 지속적으로 관리한다.

■ 자료 관리 계획 개발·수정·보완

관리필요성	체계적인 자료 관리를 위해 차기 연도 자료 관리 계획을 연구 시작 시점에서 수립하는 것이 바람직하다.
관리 방법	매년 하반기 당해 자료 질 관리 전략 및 수행 정도를 기반으로 계획서를 개발하고, 연속되는 진행사항에 대하여 수정 및 보완하도록 한다.

■ 통계 지표 산출

관리필요성	질 높은 연구 성과물 도출을 위해 성과활용을 위한 통계지표를 개발하고, 수집된 자료를 활용할 수 있는 방법에 대해 개발하여, 활용 가능한 다양한 연구 주제를 파악할 수 있다.
관리 방법	매년 자료의 기술 통계량을 확인하고, 향후 연구 성과물에 활용할 수 있도록 당해 연도 자료와 전체 연구 기간 동안 수집된 자료를 이용하여 통계지표를 산출한다.

3) 각 코호트 연구별 데이터 전략 수립 시 주의사항

(1) 데이터 품질관리 과정을 지속적으로 수행되어야하며 각 단계별 계획을 수행한 이후에는 반드시 적용·수정 여부에 대하여 검토한다.

(2) 위 과정은 표준화된 절차를 나타낸 것으로 각 연구별 데이터 품질관리 전략을 수립하는데 참고자료로 활용이 가능하나, 연구 설계에 따른 조사 방법이 다른 경우 반드시 예측 허용 범위를 벗어나는 관측 값에 대한 확인과 보정을 별도로 수행한다.

(3) 품질관리주기는 항목에 따라 다르므로, 매년 일정을 고려하여 계획을 수립한다.

4 데이터 품질관리 지침서 작성 기준

1) 조사서 및 조사지침서

(1) 포함내용

연구에서 사용하는 조사서(종이조사서, 전자조사서)는 그 형태가 일치해야하며, 주요 항목별 조사 문항이 일관성 있어야한다. 조사지침서는 조사서의 조사 문항별 지침에 대해 상세히 기록되어 있어야 한다.

(2) 작성기준

■ 조사서

조사 항목의 순서는 대상자와 조사원 모두가 조사에 어려움이 없도록 구성되어야 하며, '모름', '기타'와 같이 대상자의 주관적 판단에 의해 응답이 결정되는 선택지를 최소화한다. 조사원과 대상자가 조사서를 이해하는데 어려움이 없도록 한자어와 전문용어보다는 대중적으로 사용하는 용어를 사용한다.

■ 조사지침서

표준화된 지침은 문항별로 알기 쉽게 정리하여 표기하고, 조사에 필요한 내용을 모두 포함한다. 조사원이 적절한 응답을 유도하고 대상자의 응답을 조사서 형식에 맞춰 기입할 수 있도록 예시를 제시하고, 수정·보완 시에는 이전의 지침과 새로운 지침을 확인할 수 있도록 기록으로 남긴다. 수정된 지침이 결정된 회의와 시기(년/월)를 명시하고, 이후 해당 문항에 대한 재논의가 있더라도 결정 내용의 변경 사항을 확인할 수 있도록 한다. 이외에 조사에 참고할 만한 사항은 부록으로 첨부하여 연구자 혹은 조사원이 조사 시 참고할 수 있도록 한다.

2) 원시자료이용지침서

(1) 포함내용

실제로 데이터를 사용할 연구자가 자료를 이해하기 쉽도록 조사개요(목적, 대상, 수행체계, 조사 방법 및 도구, 조사서 변화 체계)와 원시자료의 구성(변수별 조사서 변경 과정을 기록), 변수 설명서(조사서와 코드북이 포함된 형식) 순으로 작성한다. 필요에 따라 본문에 포함되지 못한 주요 내용을 부록으로 별첨한다.

(2) 작성기준

원시자료이용지침서는 조사서 내 조사 항목 순으로 정리하고, 조사 변수에 대한 간략한 설명과 다운로드 받은 데이터 안에 정의된 코드 값을 함께 명시한다. 원시자료이용지침서는 가장 최근의 조사서를 기반으로 작성하는 것을 원칙으로 하나, 과거에 수집하다가 현재 더 이상 수집하지 않는 변수가 다운로드 자료에 포함된다면 해당 변수에 대한 설명도 포함해야한다.

3) 코드북

(1) 포함내용

조사서와 다운로드 받은 자료의 약속된 코드 값을 정리한 내용으로 변수를 수집한 조사서의 버전을 포함하여 데이터베이스에 적용된 로직에 대해서도 명시되어야 한다. 수정·보완 시에는 조사지침서와 같이 그 내용을 모두 기록으로 남긴다.

(2) 작성기준

코드북을 주로 사용하는 연구원은 역학팀 연구원과 데이터베이스 고도화 작업을 수행하는 전산팀이다. 약속된 코드 값을 파악하고 지속적으로 수정·보완해야하기 때문에 각 연구팀에서 자료를 가장 잘 다루는 사람이 담당하며, 자료 내 연구 시작 이후 최근까지 수집된 전 변수(다운로드 시 포함되는 모든 변수)의 조사유형, 한글변수명, 영문변수명, 조사주기, 조사서버전, 변수의 속성과 유형, 약속된 코드 값(최소 허용 값, 최대 허용 값, 결측 값), 논의사항을 모두 기록으로 남긴다.

4) 자료 관리 계획서

(1) 포함내용

권장하는 데이터 품질관리 전략을 기반으로 각 연구의 특성이 고려된 데이터 품질관리 방법과 계획에 대한 내용을 포함한다. 뿐만 아니라 연구에서 사용하는 자료보안기준과 담당자를 명시하고, 관련 문의가 있을 경우 이를 참고하여 문제를 해결한다. 자료 관리 중 발생한 문제를 해결하고 자료 관리방법을 제시하여 담당자 부재 시에도 문제를 해결할 수 있도록 한다. 자료 관리 계획서에서는 정제가 완료된 자료의 분양 기준을 표준화된 자료 정제율을 기반으로 표기한다. 더불어 결측 값에 대한 처리 계획 및 방안에 대하여 기술한다.

(2) 작성기준

연구의 특성을 고려하여 실제 수행되는 계획에 따른 관리 방법을 제시한다. 자료 관리는 체계적으로 수행되어야 하므로 시간에 따른 순서 혹은 중요도 순으로 작성한다. 자료 관리 계획에 대한 별도의 결과보고서는 없으나, 매년 연구 종료 시점에 차기 년도 자료 관리 계획을 수립하면서 수행정도에 따른 추가 계획 등을 함께 명시하는 것을 권장한다. 즉, 실제 연구를 수행하며 자료 관리를 위한 실무 내용을 단계적으로 파악할 수 있도록 한다.

5) 로직북

(1) 포함내용

자료 정제 과정에서 사용되는 논리적 오류(로직, logic)를 규칙에 의해 정의된 로직 번호, 로직 종류, 해당 회차, 그리고 변수(항목, 세부항목, 한글변수명, 영문변수명, 조건변수명)와 오류 내용을 포함하여 정리한다.

(2) 작성기준

마지막 정제 시 적용된 로직을 기준으로 하며, 경우에 따라 데이터 베이스에 적용된 로직에 대해서는 DB 로직 적용 여부를 표기하고 관련 내용에 예시 및 참고사항을 추가로 표기하여 기록으로 남긴다. 정제용 데이터를 별도로 구축하였다면 데이터의 이름을 함께 표기하여 추후 확인 가능하도록 한다.

5 연구 담당자

1) 만성감염질환 코호트 데이터 품질관리 표준화 지침 개발

이름	소속	역할	참여형태
최보율	한양대학교 의과대학	총괄/HIV/AIDS 코호트	책임연구원
기모란	국립암센터	HCV 코호트/HPV 코호트	공동연구원
김범경	연세대학교 신촌세브란스병원	HBV 코호트	공동연구원
박보영	한양대학교 의과대학	총괄	공동연구원
최윤수	한양대학교 의과대학	총괄/HIV/AIDS 코호트	연구원
김민정	한양대학교 의과대학	HIV/AIDS 코호트	연구원
백다혜	국립암센터	HCV 코호트	연구원
최화영	국립암센터	HCV 코호트	연구원
박윤	국립암센터	HPV 코호트	연구원
김병우	국립암센터	HPV 코호트	연구원
김수민	한양대학교 의과대학	HIV/AIDS 코호트/HBV 코호트	연구원
이재승	연세대학교 신촌세브란스병원	HBV 코호트	연구원
