# Supplementary material for: Prospective cohort data quality assurance and quality control strategy and method: Korea HIV/AIDS Cohort Study
Source: Epidemiol Health. 2020 Sep 4;42:e2020063. doi: 10.4178/epih.e2020063 (PMC7871148; doi:10.4178/epih.e2020063)
Supplement: Supplementary file 2 [file epih-42-e2020063-suppl2.docx]

**Prospective cohort data quality assurance and quality control strategy and method: Korea HIV/AIDS Cohort Study**

**Strategies for enhancement of data quality control**

**First author:** Soo Min Kim^1), 2), 3)^, Yunsu Choi^3), 4)^

**Soo Min Kim, Yunsu Choi, Bo Youl Choi,** Minjeong Kim^3)^, Sang il Kim^5)^, Jun Young Choi^6)^, Shin-Woo Kim^7)^, Joon Young Song^8)^, Youn Jeong Kim^9)^, Mee-Kyung Kee^10)^, Myeongsu Yoo^10)^, Jeong Gyu Lee^10)^, Bo Young Park^4)^

1. Department of Applied statistics, College of Commerce and Economics, Yonsei University, Seoul, Korea
2. Department of Statistics and Data Science, College of Commerce and Economics, Yonsei University, Seoul, Korea
3. Institute for Health and Society, Hanyang University, Seoul, Korea
4. Department of Preventive Medicine, Hanyang University College of Medicine, Seoul, Korea
5. Division of Infectious Disease, Department of Internal Medicine, Seoul St. Mary's Hospital, College of Medicine, The Catholic University of Korea, Seoul, Korea
6. Department of Internal Medicine and AIDS Research Institute, Yonsei University College of Medicine, Seoul, Republic of Korea
7. Department of Internal Medicine, School of Medicine, Kyungpook National University, Daegu, Korea
8. Division of Infectious Diseases, Department of Internal Medicine, Korea University College of Medicine, Seoul, Korea.
9. Division of Infectious Disease, Department of Internal Medicine, Incheon St. Mary's Hospital, College of Medicine, The Catholic University of Korea, Incheon, Korea
10. Division of Viral Disease Research Center for Infectious Disease Research, Korea National Institute of Health, Cheongju-si, Korea

**Correspondence:** Bo Youl Choi

Department of Preventive Medicine, Hanyang University College of Medicine, 222 Wangsimni-ro Seongdong-gu, Seoul 04763, Korea

E-mail: [bychoi@hanyang.ac.kr](mailto:bychoi@hanyang.ac.kr)

ABSTRACT

**Objectives:** The aim of effective data quality control and management is to minimize the impact of errors on study results by identifying and correcting them. This study presents the results of a data quality control system for the Korea HIV/AIDS Cohort Study that took into account the characteristics of the data.

**Methods:** The HIV/AIDS Cohort Study in Korea conducts repeated measurements every 6 months using an electronic survey administered to voluntarily consenting participants and collects data from 21 hospitals. In total, 5,795 sets of data from 1,442 participants were collected from the first investigation in 2006 to 2016. The data refining results of 2015 and 2019 were converted into the data refining rate and compared.

**Results:** The quality control system involved 3 steps at different points in the process, and each step contributed to data quality management and results. By improving data quality control in the pre-phase and the data collection phase, the estimated error value in 2019 was 1,803, reflecting a 53.93% reduction from 2015. Due to improvements in the stage after data collection, the data refining rate was 92.68% in 2019, a 24.21%p increase from 2015.

**Conclusions:** Despite this quality management strategy, errors may still exist at each stage. Logically possible errors for the post-review refining of downloaded data should be actively identified with appropriate consideration of the purpose and epidemiological characteristics of the study data. To improve data quality and reliability, data management strategies should be systematically implemented.

KEY WORDS. HIV/AIDS, quality control, Cohort Studies, data adjustment, Data Quality, data accuracy

1. **서론**

경시적 자료는 동일한 대상을 장기간 추적 관찰하여 특정 요인에 대해 반복적으로 조사한 자료이다. 경시적 자료의 한 종류인 코호트 자료는 특정 위험 요인에 노출된 인구 집단과 노출되지 않은 인구 집단을 장기간 추적 관찰하여 두 집단의 질병의 발생률이나 사망률을 비교하는 역학 연구 설계로 원인과 결과에 대한 시간적 선후 관계가 명확하다는 장점이 있다.

코호트 연구에서 확인되는 편향(bias)으로는 자발적으로 연구 참여에 동의한 사람과 그렇지 않은 사람의 특성 차이에 의하여 발생하는 자발적 참여자 편향(volunteer bias), 조사 참여 도중 사망이나 탈락으로 인해 발생하는 추적관찰 탈락 편향(follow-up loss bias), 노출 군과 비노출 군에서 질병 정보의 조사 과정이 다르기 때문에 발생하는 확인 편향(ascertainment bias), 위험 요인의 반복 측정에 의한 대상자의 행동 변화에 따른 호손 효과 (Hawthorne effect), 추적 기간에 따라 진단 기준이나 대상자의 개인적 요인이 변화하여 영향을 받는 시간 편향(time bias) 등이 있다. 그 외 대부분의 연구 디자인에서 공통으로 가지고 있는 편향으로는 조사 문항에 응답 값이 없는 무응답 편향(non-response bias), 면담자와의 조사 과정에서 발생할 수 있는 정보 편향으로 면담자 편향(interviewer bias), 측정 편향(measurement bias)가 존재할 수 있다. 또한, 많은 자료의 오류는 연구를 수행하는 과정에서 예기치 않게 우연히 발생한다 [1]. 이에 자료 수집 전부터 자료가 구축되는 시점까지의 전 과정에서 적절한 질 관리를 통해 통제될 필요가 있다.

질 관리는 모든 연구를 수행하는 데 있어 가장 중요하며, 연구 결과의 무결성은 수집된 연구 자료의 질에 의해 결정된다. 코호트 자료를 활용하는 데 적절한 질 관리가 이뤄지지 않으면 검정력(1-β)이 낮아지고, 제1종 오류(α)를 증가시킬 수 있다 [2]. 연구 자료의 질에 영향을 주는 주요인으로는 완성도(Completeness)와 정확도(Accuracy), 그리고 적시성(timeliness)이 있고 [3], 이를 위해서는 데이터의 정확한 입력, 입력된 데이터의 모니터링, 데이터 클리닝 등이 단계적으로 필요하다 [2, 4-7]

국내 신고자료에 따르면 2018년까지 사망한 감염인을 제외한 생존 감염인 수는 12,991명(25.3/100,000명)이고, 2017년까지 인구 10만 명 당 AIDS 발생은 0.3명으로 OECD 평균에 비해 낮다 [8, 9]. 한국 HIV/AIDS 코호트 연구는 국내 HIV 감염인과 환자들의 초기 진단에서 AIDS 발병과 사망에 이르기까지의 자연사와 역학적 임상적 특성을 파악하기 위해 구축되었다. 이 연구는 Western blot 검사에 의해 HIV 양성 확진을 받은 만 18세 이상의 연구 참여 병원의 내국인 감염인에 한하여 자발적으로 동의한 대상자만이 참여하고 있다 [10]. 2019년 10월까지 1,539명의 16,830건의 조사서가 수집되었고, 이들의 일반적 특성, 사회경제적요인, 동반상병(AIDS 관련, AIDS 비 관련), ART 치료력, 종결 사유 등에 대한 조사는 6개월 단위로 반복 측정되고 있다 [10, 11]. 이렇게 수집된 자료는 한국 HIV/AIDS 코호트 연구에 참여하는 연구자들이 HIV/AIDS 관련 중개연구 수행을 위한 연구에 활용하고 있다. 연구의 목적은 실제 임상 현장에서 환자에게 도움이 되는 치료 및 예방법의 근거로 사용하기 위함이고, 질 관리가 제대로 되지 않은 연구 결과는 실제(real world)에 적용하기 어렵다.

이와 같이 HIV 관련 연구 가설을 검증하고 질 높은 연구 결과를 도출하기 위해서는 제대로 된 질 관리가 필요하다. 이 연구에서는 기존의 자료 질 관리 전략을 기반으로 국내 HIV/AIDS 코호트 연구 자료의 특성을 고려한 맞춤형 질 관리 전략을 적용하여 과거(2015년 10차 연구)와 최근(2019년 14차 연구)의 자료 정제율(rate of data cleaning)을 비교해 객관적으로 자료 질 관리 효과를 판단하고자 한다.

1. **연구 방법**
   1. **Epidemiology and data center**

한국 HIV/AIDS 코호트 연구는 전국 21개 병원의 감염내과에서 자료를 수집하고 자료의 질 관리를 담당하는 역학데이터센터가 있다. 역학데이터센터 내에는 조사와 관련된 내용을 담당하는 역학팀과 자료 정제 과정을 담당하는 질 관리팀, 그리고 연구 분석을 지원하는 통계분석팀이 존재한다 [10].

- 1. **Data resource**

한국 HIV/AIDS 코호트 연구는 자발적으로 동의한 대상자에 대해 전자조사서를 이용하여 6개월 단위로 반복 측정을 시행하며, 21개 기관에서 실시간으로 자료를 수집한다. 2006년 최초 조사를 시작하여 2016년까지 1,442명에 대한 조사서 5,795건이 수집되었다 (Appendix A).

- 1. **Data quality assurance and quality control protocol**

한국 HIV/AIDS 코호트 연구 자료 질 관리 전략(프로그램)의 전 과정은 크게 자료 수집 전 단계, 자료 수집 단계, 자료 수집 후 단계로 구분된다. 각 단계별 구체적인 전략과 종류, 관리 주기, 방법 그리고 미 시행 시 발생 가능한 오류를 제시하였다. 자료 수집 전 단계에서는 조사서 수정과 보완, 조사원 표준화 교육, DB와 조사서, 코드북 등의 값을 일치시키는 DB 코드 일원화 작업, 논리적 오류 도출을 위한 로직 개발과 표준화된 조사지침서 개발을 시행하고, 자료 입력 단계에서는 실시간 모니터링, 반복조사율 관리, 데이터 베이스 질의어(data query language) 관리를 통해 자료 입력 시점에 발생 가능한 오류를 최소화시킨다. 자료 수집 후 단계에서는 개발한 논리적 오류 로직을 이용해 자료정제(Data cleaning)를 수행하고, 결측값을 최소화하기 위한 재조사, 서술형 문항 표준화 등으로 자료의 정확도를 높인다. 모든 과정의 자료 검토에는 감염내과 전문의의 검토가 포함되고, 자료의 오류 예상 값을 도출하는 정제 과정에서는 2인 이상의 역학통계연구원이 중복 검토를 시행하며, 수정된 자료가 올바르게 데이터 베이스에 입력되었는지 검토하는 과정에서는 역학데이터센터와 질병관리본부 데이터베이스 담당자가 중복으로 검토를 시행한다(Figure 1, Table 1).

자료를 활용한 연구 분석 시 중요 문항의 결측값이 많은 경우에는 다른 값으로 대체하여 자료의 완성도를 높인다. 자료 수집 전 단계와 자료 수집 단계에서의 질 관리를 시행한 후에도 결측값이 있을 때는 가능하다면 다른 값으로 추정을 하며 추정 방법은 다음과 같다.

1. 공식을 이용한 날짜 대체

단순히 날짜에 부분 결측이 있을 때는 조사일을 이용하여 다음과 같이 추정한다.

$$\mathbf{New HIV diagnosis month (day)=}\mathbf{survey}\mathbf{month(day)-}\frac{\mathbf{survey month(day)}\boldsymbol{-1}}{\boldsymbol{2}}$$

1. 내부 자료를 이용한 대체

기존 자료에서 특정 시점 당시 자료가 없는 경우 특정 시점 전후 90일 내 가장 가까운 시점의 자료로 대체한다. 실제 자료에서 진단 시점이나 최초 치료 시작일의 면역 검사 결과가 결측일 때, 진단 시점 혹은 최초 치료 시작일 전후의 가장 가까운 시점의 검사 결과로 대체한다. 진단 시점 전후에서 인정하는 기간을 증가시킬수록 대체율은 높아졌으나 6개월 단위의 반복 조사 주기를 고려하여 3개월로 정의하였다.

$$\boldsymbol{Blood test date -HIV diagnosis date (Initial ART date) \leq3 months}$$

1. 외부 자료를 이용한 대체

HIV 양성 확진일, 사망일, 감염경로(수혈, 혈액 응고 제제)가 결측 값일 때, 질병관리본부 HIV/AIDS 신고 자료 내에 포함된 자료를 이용하여 이들을 대체한다.

- 1. **자료 질 관리 전략의 실제 데이터 적용 결과**

위와 같이 개발한 자료 질 관리 전략 중 자료 수집 후 단계에서의 질 관리 방법을 한국 HIV/AIDS 코호트의 10차 연도와 14차 연도 데이터에 적용하여 자료 정제율을 비교해 보았다. 자료 정제율을 산출하기 위해서는 3가지 요인이 필요하다. 3가지 요인에는 개발된 로직에 의해 오류라고 추정되는 오류 예상 값(expected errors to be handled)이 필요하고, 오류 예상 값을 각 조사 기관에 확인을 의뢰하여 실제 오류로 판명되어 수정된 오류 값(corrected errors), 그리고 로직에 의해 오류로 분류되었으나 의무기록을 확인하였을 때 오류가 아닌 실제로 관측된 값(non-errors)이 포함된다. 이 세가지 요인을 활용하여 조사 기관으로 확인을 요청한 오류 예상 값의 수(분모) 대비 수정 및 확인된 오류(수정된 오류 값과 실제 값의 합, 분자)를 백분율로 환산한 값이 정제율로 정의된다. 즉, 정제율은 추정된 오류 예상 값이 수정된 비율을 의미하며, 정제율이 높을 수록(100에 가까울 수록) 확인 가능한 오류가 정제율만큼 수정되었음을 의미한다.

$$Rate of data cleaning\left( \% \right)=\frac{corrected errors + non errors}{expected errors to be handled} \times100$$

1. **연구 결과**

자료 질 관리 전략을 통해 조사서 5,795건과 5,027개의 조사 문항으로 이루어진 약 2,900만개의 자료를 정제한 결과 14차년도 연구에서의 오류 예상값은 1,803개로, 10차년도 연구 오류 예상값 대비 53.9% 감소하였다. 이는 자료 질 관리 전략의 단계 중 자료 수집 전 단계의 DB 코드값 일원화, 표준화 조사 지침 마련 및 교육, 조사서 수정 및 보완 및 자료 수집 단계의 자료 입력 관리, DB 로직 개발을 체계적으로 수행하여 얻어진 결과이다. 10차 년도 오류 예상 값 도출 전에는 체계적인 자료 질 관리 전략이 구축되지 않아, 단계적으로 수행하는 질 관리 절차 없이 필요에 따라 표준화 교육이나 신규 연구 조사원의 조사서 입력 값에 대해서만 모니터링을 일시적으로 수행하였다. 하지만 체계적인 절차를 구축함에 따라 실시간으로 수집되는 모든 조사서를 모니터링하고, 주기적으로 표준화 교육을 정규, 신규, 보수로 구분하여 진행하는 등의 방법을 통해 산발적으로 발생하는 오류 예상 값을 대폭 감소 시킬 수 있었다.

한편 자료 수집 단계 시점까지의 체계적인 질 관리 전략에도 불구하고, 자료 수집 후 단계에서 수집된 자료를 확인했을 때 타 병원에서의 진료 및 처방 기록에 대한 확인이 어렵거나, 대상자 자체가 응답을 거부한 경우 등의 조사 과정에서 발생하는 결측 값이 존재하게 된다. 반복 측정 자료에서 결측 값은 연구 결과에 영향을 미칠 수 있기 때문에 이를 보완하는 방법이 필요하다. 기존 연구에서는 통계적 방법에 의한 대치(Imputation)방법을 사용하나 이는 단순히 수리통계학적 이론에 근거한 것으로 임상적 특성을 충분히 고려하지 못한다는 한계가 있다. 우리 연구에서는 이를 보완하기 위해 진단 시점 혹은 최초 치료 시작일 시점의 CD4나 HIV RNA에 결측 값이 있을 때, 진단 시점 전후 기간이 1개월 혹은 1년 이내의 검사 결과로 대치해 비교하였다. 그 결과 CD4 및 HIV RNA 결괏값이 진단일 기준 검사 시행일이 1개월 내외에 존재하는 경우 38.8%, 35.1%에 불과한 반면, 1년으로 범위를 확장 시켰을 때 73.68%, 71.01%로 증가하였다. 최초 치료 시작 일을 기준으로 면역 검사 결과를 정의하였을 때에도 동일하였다(Table 2). HIV 양성 확진일의 경우 11.9%에서 결측이 있었는데, 이 중 11.5%는 질병관리본부 HIV/AIDS 신고 자료를 이용하여 대치할 수 있었고 0.4%는 두 자료 모두에서 양성확진일을 확인할 수 없었다. 이와 같이 특정 시점의 자료가 결측인 경우 내부, 외부 자료를 이용하여 대치한 후 연구 활용을 위한 자료 완성도를 높인다.

또한 앞선 체계적인 질 관리 전략의 수행에도 불구하고, 자료를 다운로드 하였을 때 여전히 오류가 남아있는 것을 확인할 수 있다. 이는 자료 수집 후 단계에서 정제용 로직을 이용해 오류 예상 값(expected errors to be handled)으로 도출된다. 10차년도 연구에서는 2006년 12월부터 2014년 12월까지 수집된 자료를 정제하여 오류 예상값 3,914개를 도출하였고, 이 중 629개의 실제값을 제외한 2,051개의 오류를 수정하여 68.47%의 정제율로 자료를 정제하였다. 14차년도 연구에서는 2006년 12월부터 2019년 07월까지 수집된 자료에 대한 정제 작업을 시행하여 오류 예상값 1,803개를 도출하였고, 이 중 397개의 실제값을 제외한 1,274개의 오류를 수정하여 92.68%의 정제율로 자료를 정제하였다.

정제율을 기준으로 14차년도 자료의 질이 10차년도에 비해 약 24.2%p 증가하였다(Table 3). 한국 HIV/AIDS 코호트 연구는 이러한 정제 과정을 통해 같은 기간 동안 수집된 자료를 2회에 걸쳐 정제하고, 정제율이 90% 이상이면 해당 기간의 자료의 질이 공개 분양 가능하다고 판단하고 해당 기간 수집된 자료의 데이터 베이스는 임의로 수정할 수 없도록 잠근다. 잠긴 자료는 자료관리 책임자를 통해서만 수정가능하게 되며 모두 기록으로 남겨 자료 질 관리를 유지한다. 정제가 완료된 자료는 코호트 운영위원회를 통해 분양 여부를 결정하며, 배포용 자료로 결정되면 "질병관리본부 원시 자료 공개 절차 등에 관한 규정"에 따라 내외부 연구자에서 분양을 시행한다. 코호트 내부연구자들은 연구계획서를 질병보건통합시스템(http://is.cdc.go.kr)에 제출 후 코호트 운영위원회 평가 및 승인을 받은 후 제반 규정에 의해 자료를 분양받는다. 외부연구자의 경우는 질병보건통합시스템에 연구계획서가 제출되면 연구계획 평가위원회가 개최되며, 평가결과에 의거한 질병관리본부 바이러스질환연구과장의 최종 승인 후 자료를 사용할 수 있게 된다. 이후 자료 사용 일정을 조율 한 뒤, 질병관리본부 바이러스질환연구과 학술연구용 자료분석실에서 자료 분석이 가능하며, 결과는 검토 승인 이후 반출할 수 있다. 자료 사용에 대한 결과물은 질병관리본부 연구 성과물 규정을 준수하여야 한다. 자세한 내용은 질병관리본부 통합관리시스템에서 확인할 수 있다.

1. **요약 혹은 DISCUSSION**

연구에서의 실질적인 데이터 활용과 연구를 통한 국내 HIV/AIDS 감염인들의 삶의 질 개선을 위해서는 데이터 수집과 정제에 참여하는 모든 사람들의 노력이 필요하다. 데이터를 활용하여 연구 성과를 내는 것도 중요하지만 정확한 분석을 하기 위해서는 정확한 데이터가 선행되어야 한다. 한국 HIV/AIDS 코호트 연구에서는 코호트 대상자와 자료의 특성을 반영한 국내 최초의 코호트 맞춤형 자료 질 관리 전략을 구축하였고, 코호트 자료의 완결성을 높이는 방법에 대해 제시한다. 기존 연구에 따르면 코호트 자료 질 관리 전략은 대부분 자료 수집 시점까지의 단계에서 이루어지고 있다. 하지만 이 연구에서는 자료 수집 시점까지 뿐만 아니라 수집 이후의 단계에서 질병의 임상 및 역학적 특성을 고려한 논리적 오류를 발견하여 수정함으로써 이전 단계에서 확인하기 어려운 여러 문항 및 회차 사이의 오류를 집중적으로 도출할 수 있다. 또한 코호트 자료에 HIV/AIDS의 임상역〮학적 근거와 통계적 방법을 결합한 논리적 오류를 적용하여, 전산상 단순한 오류로 도출되지 않지만 임상역〮학적 가설에 어긋나는 오류 값을 사전에 도출하여 정제할 수 있다. 실제로 연구 주제에 따라 여러 문항을 복합적으로 사용하여 대상자를 정의하거나 기존에 수집된 자료를 활용하여 연구자의 조작적 정의에 의해 연구용 변수를 생성할 때 오류가 발생할 수 있다. 연구자가 이러한 오류를 연구 수행 과정 중에 발견하게 되어도 자료를 직접 확인할 수 없기 때문에 연구 활용에 제한이 생기고, 이러한 오류를 파악하지 못한 상태로 연구가 수행되면 연구 결과 해석에 편향(bias)이 생길 수 있다. 따라서 논리적 오류는 연구 자료의 수집 목적, 질병의 임상 및 역학적 특성, 그리고 대상자의 특성을 고려하여 개발될 필요가 있다.

이 연구가 제시하는 질 관리 전략은 체계적인 단계로 구성되어 있고, 각 단계는 자료 질 관리에 복합적으로 기여한다. 따라서 어느 한 단계의 과정이 누락되거나 잘못 시행되지 않는 것이 중요하다. 예를 들어, CRF 수정 및 조사자 교육, 그리고 각종 지침서를 개발하는 등의 자료 수집 전 단계의 과정이 누락될 경우 변화하는 HIV/AIDS 역학적 특성을 연구에 적용하기 어렵고, 자료 수집 및 활용에 대한 서식이 표준화되지 않으면 자료 수집 및 활용에 제한이 생긴다. 또한 자료 수집 단계의 과정이 누락되면 실시간으로 수집되는 전자 조사서의 오류를 파악하기 어렵다. 만약 전자 조사서의 오류로 인해 잘못 입력되거나, 다운로드 오류가 발생하는 등의 문제를 해결하지 못한다면 수집된 자료의 신뢰 및 타당도가 감소한다. 또 자료 수집 후 정제 과정이 누락되면 이전 단계에서 확인하지 못한 여러 회차 및 문항 사이에 발생하는 오류를 확인할 수 없다.

이와 같이 단계적인 자료 질 관리 전략은 연구 결과에서 보듯이 10차 연도 대비 14차 연도 연구에서 자료 수집 전 단계, 자료 수집 단계의 개선으로 오류 예상값을 감소시키고, 자료 수집 후 단계의 개선으로 인해 정제율을 증가시켰다. 현재까지 역학연구자료를 활용한 질적 검토 방법이나 전략으로 ‘정제율’과 같이 결과를 제시하는 표준화된 용어는 없었다. 임상 자료를 활용한 연구에서도 자료 정제 과정(flow)에만 초점을 두었고, 정제의 상세한 단계나 과정의 결과에 대한 연구는 없었다. 정제율이 68.4% 라는 것은 오류 예상 값으로 도출된 1,000개의 오류 중에 684개의 오류 예상 값이 수정되었고, 나머지 316개의 오류는 여전히 오류로 남아있다는 의미이다. 정제율의 증가는 남아 있는 오류 예상 값이 감소하였다는 것을 의미하므로, 이 자체로 자료의 신뢰도가 전에 비해 개선되었다고 할 수 있다.

자료의 질은 연구 분석 결과에도 영향을 미친다. 보다 더 나은 질의 자료와 신뢰도 높은 연구 결과를 위해서는 앞으로도 자료 질 관리 전략을 체계적으로 잘 시행하는 것이 중요하다. 이와 같이 체계적이고 복잡한 정제 과정을 시행함에도 모든 오류와 결측 값을 ‘0’으로 처리하는 데에는 한계가 있다. 코호트 자료 특성 상 추적율을 높여 결측값을 줄이고, 근본적으로 전자 조사서 입력 시점의 오류 발생을 방지할 필요가 있고, 여러 단계를 통해 자료 수집 후 처리 과정을 최소화해야한다. 향후 기술의 발전으로 데이터 질의어(data query language)로 전자조사서 입력 시점의 오류를 방지할 수 있게 되더라도 다운로드 자료에 대한 사후 검토는 반드시 시행되어야 한다. 이러한 이유에서 개발된 논리적 오류 이외에 잠재되어 있는 오류 값이 존재할 수 있고, 정제가 완료된 자료가 오류가 없는 완벽한 자료라고 말할 수는 없기 때문에 논리적 오류에 대한 꾸준한 개발 및 보완이 필요하다.

1. **참고 문헌**

1.Van den Broeck J, Cunningham SA, Eeckels R, Herbst K. Data cleaning: detecting, diagnosing, and editing data abnormalities. PLoS Med 2005;2:e267.

2.Whitney CW, Lind BK, Wahl PW. Quality assurance and quality control in longitudinal studies. Epidemiologic reviews 1998;20:71-80.

3.Chen H, Hailey D, Wang N, Yu P. A review of data quality assessment methods for public health information systems. International journal of environmental research and public health 2014;11:5170-5207.

4.Gassman JJ, Owen WW, Kuntz TE, Martin JP, Amoroso WP. Data quality assurance, monitoring, and reporting. Controlled clinical trials 1995;16:104-136.

5.Schmidt MI, Griep RH, Passos VM, Luft VC, Goulart AC, Menezes GMdS, et al. Strategies and development of quality assurance and control in the ELSA-Brasil. Revista de saude publica 2013;47:105-112.

6.Teppo L, Pukkala E, Lehtonen M. Data quality and quality control of a population-based cancer registry: experience in Finland. Acta oncologica 1994;33:365-369.

7.Pandav R, Mehta A, Belle SH, Martin DE, Chandra V, Dodge HH, et al. Data management and quality assurance for an International project: the Indo–US Cross‐National Dementia Epidemiology Study. International journal of geriatric psychiatry 2002;17:510-518.

8. Korea Centers for Disease Control. Korea notification of HIV/AIDS in 2018 [cited 2020 Aug 05]. Available from: <http://www.cdc.go.kr/npt/biz/npp/portal/nppPblctDtaView.do?pblctDtaSeAt=1&pblctDtaSn=1939>

9.정상운, 차정옥, 윤재규, 공인식. 2018 년 HIV/AIDS 신고 현황. 주간 건강과 질병 2019;12:1182-1188.

10.Choi BY, Choi JY, Han SH, Kim SI, Kee M-K, Kim MJ, et al. Korea HIV/AIDS Cohort Study: study design and baseline characteristics. Epidemiology and health 2018;40

11.Choi Y, Choi BY, Kim SM, Kim SI, Kim J, Choi JY, et al. Epidemiological characteristics of HIV infected Korean: Korea HIV/AIDS Cohort Study. Epidemiology and Health 2019;41
